# Supplementary material for: TastepepAI: An artificial intelligence platform for taste peptide de novo design
Source: PLoS Comput Biol. 2025 Oct 16;21(10):e1013602. doi: 10.1371/journal.pcbi.1013602 (PMC12543283; doi:10.1371/journal.pcbi.1013602)
Supplement: S1 File — (ZIP) [file pcbi.1013602.s006.zip › Supplementary Data 2/TaPep9 MS.doc]

# Mass Spectrometry Report

|   [M+H]+  [2M+H]+ | | | | | | | |
| --- | --- | --- | --- | --- | --- | --- | --- |
|  | Sample Description | | Instrument | SHIMADZU LCMS-2020 | | | |
|  | Analyzed date: | 2024-9-25 | Probe: | ESI | | Probe Bias: | ＋4.5kv |
|  | Analyst: | SHAO | Nebulizer Gas Flow: | 1.5L/min | | Detector: | 1.5kv |
|  | Sample: | FCL-NJP93310 TaPep9 VA-6 | CDL: | -20.0v | | T. Flow: | 0.2ml/min |
|  | M.W.: | 657.75 | CDL Temp.: | 250 | ^o^C | B. Conc.: | 50%H_2_O/50%ACN |
|  | Lot. No.: | P240913-MX1172213 | Block Temp.: | 200 | ^o^C |  |  |
